# Multipoint genome-wide linkage scan for nonword repetition in a multigenerational family further supports chromosome 13q as a locus for verbal trait disorders

**DOI:** 10.1007/s00439-016-1717-z

**Published:** 2016-08-17

**Authors:** D. T. Truong, L. D. Shriberg, S. D. Smith, K. L. Chapman, A. R. Scheer-Cohen, M. M. C. DeMille, A. K. Adams, A. Q. Nato, E. M. Wijsman, J. D. Eicher, J. R. Gruen

**Affiliations:** 1 Department of Pediatrics, Yale School of Medicine, New Haven, CT 06510 USA; 2 Waisman Center, University of Wisconsin-Madison, Madison, WI 53705 USA; 3 Department of Pediatrics, University of Nebraska at Omaha, Omaha, NE 68182 USA; 4 Department of Communication Sciences and Disorders, University of Utah, Salt Lake City, UT 84112 USA; 5 Department of Speech-Language Pathology, California State University, San Marcos, CA 92096 USA; 6 Department of Genetics, Yale School of Medicine, New Haven, CT 06510 USA; 7 Division of Medical Genetics, Department of Medicine, University of Washington, Seattle, WA 98195 USA; 8 Department of Biostatistics and Department of Genome Sciences, University of Washington, Seattle, WA 98195 USA; 9 Investigative Medicine Program, Yale School of Medicine, New Haven, CT 06510 USA

## Abstract

Verbal trait disorders encompass a wide range of conditions and are marked by deficits in five domains that impair a person’s ability to communicate: speech, language, reading, spelling, and writing. Nonword repetition is a robust endophenotype for verbal trait disorders that is sensitive to cognitive processes critical to verbal development, including auditory processing, phonological working memory, and motor planning and programming. In the present study, we present a six-generation extended pedigree with a history of verbal trait disorders. Using genome-wide multipoint variance component linkage analysis of nonword repetition, we identified a region spanning chromosome 13q14–q21 with LOD = 4.45 between 52 and 55 cM, spanning approximately 5.5 Mb on chromosome 13. This region overlaps with SLI3, a locus implicated in reading disability in families with a history of specific language impairment. Our study of a large multigenerational family with verbal trait disorders further implicates the SLI3 region in verbal trait disorders. Future studies will further refine the specific causal genetic factors in this locus on chromosome 13q that contribute to language traits.

**Electronic supplementary material:**

The online version of this article (doi:10.1007/s00439-016-1717-z) contains supplementary material, which is available to authorized users.

## Introduction

Verbal trait disorders are comorbid, developmentally associated disorders and deficits in communication. These include clinical and subclinical disorders of speech, language, reading, spelling, and writing (Shriberg et al. 2012). Speech sound disorders (i.e., excluding dysfluency) are the most prevalent verbal trait disorders at preschool age, with an estimated population prevalence of 16 % at age 4 years (Campbell et al. 2003), decreasing to 3.8 % at age 6 years (Shriberg et al. 1999) and 3.6 % at age 8 years (Wren et al. 2012). Deficits in speech frequently co-occur with impairments in multiple domains. For example, 11–15 % of children with speech sound disorders at age 6 also have language disorder (a neurodevelopmental disorder that can affect both spoken or written language; Shriberg et al. 1999). Additionally, children with speech disorders are at higher risk for reading disability, with an estimated 18 % of children with speech disorders and 75 % of children with both speech and language disorders meeting criteria for reading disability at school age (Lewis et al. 2000).

Research in the genetics of verbal trait disorders was catalyzed by the seminal studies of the KE family, a large extended pedigree segregating verbal dyspraxia (also termed Childhood Apraxia of Speech; ASHA 2007; RCSLT 2011), suggesting autosomal dominant inheritance of a single gene mutation (Hurst et al. 1990). Genome-wide linkage showed a signal peak on chromosome 7q31.1 (Fisher et al. 1998). Further fine mapping identified a point mutation in *FOXP2* that resulted in a truncated protein and loss of function in all affected individuals, but not observed in unaffected individuals (Lai et al. 2001). *FOXP2* loss of function as a causal factor for verbal dyspraxia was further validated in unrelated individuals with severe speech impairments similar to those in the KE family (Lai et al. 2001; MacDermot et al. 2005) and has been cross-validated in a number of case studies (e.g., Rice et al. 2012; Shriberg et al. 2006). Although the KE family provided an example of a verbal trait disorder phenotype with a typical pattern of monogenic inheritance, their story is the exception rather than the norm. In fact, verbal trait disorders are generally multifactorial and associated with multiple genetic and environmental factors (Kang and Drayna 2011; Peterson and Pennington 2015).

Due to the behavioral and cognitive heterogeneity of verbal trait disorders, the use of endophenotypes—underlying phenotypic factors that are associated with or contribute to the manifestation of the disorder of interest because of shared genetic factors—have been critical to the genetic study of verbal trait disorders. One endophenotype is nonword repetition (NWR), which loads onto several cognitive processes critical for language-related ability including auditory processing, receptive language ability, and motor planning and programming (Dollaghan and Campbell 1998). NWR tasks examine the ability to process and temporarily store a novel series of meaningless units of phonological information in short-term memory, and then verbally repeat the stimuli. Such measures, which are sensitive to but not specific for any one disorder, may be more closely influenced by genetic variation than the verbal trait disorder itself.

NWR task performance has a strong genetic influence, with higher concordance among monozygotic twins compared to dizygotic twins, and heritability ranging from 0.64 to 1 (Bishop et al. 1996, 2004). Furthermore, an oligogenic-trait segregation analysis of NWR in nuclear families ascertained for reading disability estimated approximately 2.4 quantitative trait loci (Wijsman et al. 2000). A family-based linkage analysis on individuals with a family history of specific language impairment (SLI) identified a linkage peak on chromosome 16q for poor performance on NWR (SLI Consortium 2002, 2004). Follow-up family-based and population-based association studies on NWR identified *CMIP* and *ATP2C2* as candidates responsible for the linkage signal (Newbury et al. 2009). *CNTNAP2* on chromosome 7q35 was also associated with NWR in families enrolled in the SLI consortium study using a candidate gene approach after it was identified as a transcriptional binding target of *FOXP2* by chromatin immunoprecipitation (Vernes et al. 2008). In addition, CNTNAP2 was identified by fine mapping a linkage analysis signal on 7q35 conditioned on language delay in the Autism Genetic Resource Exchange sample (Alarcón et al. 2002, 2005, 2008). Taken together, NWR satisfies specific testable criteria for the objective identification of endophenotypes, supporting NWR as a credible endophenotype for verbal trait disorders (Glahn et al. 2014; Lenzenweger 2013).

With the exception of the KE family, most families with a history of language impairment show a complex pattern of inheritance with subtle differences in clinical presentation within the family. In the present study, we examined an extended six-generation family with a complex pattern of inheritance for verbal trait disorders. We chose NWR in this analysis because (1) it is a robust endophenotype for verbal trait disorders (i.e., speech sound disorder, language disorder, and developmental dyslexia); (2) is highly heritable; (3) has a Mendelian model of inheritance (in at least one study; Wijsman et al. 2000); and (4) is stable throughout an individual’s lifetime, even in those who are language recovered following impairment in childhood (Bishop et al. 1996; Shriberg et al. 2009). The latter attribute of NWR tasks is particularly important because subjects within this family range in age from 3 to 95 years, requiring a phenotype that can be ascertained and compared across all age groups. The present analysis provides strong support for chromosome 13q14–q21 as a locus that contributes to poor performance on NWR in this extended pedigree.

## Methods and materials

### Ascertainment

We studied 62 individuals from a six-generation 90-member family of European ancestry with a history of verbal trait disorders. The family was ascertained with the assistance of a family member. The 62 family members assessed included 35 females and 27 males ranging in age from 3 to 95 years. There is no evidence of consanguinity based on genealogy or unexpected high kinship coefficients within the pedigree.

Written informed consent was approved by the University of Wisconsin-Madison Institutional Review Board (IRB). All subjects were assessed by one of two experienced examiners in the participants’ homes or hotel sites in five states within the continental US. All oral instructions and audio-recorded stimuli were presented at comfortable listening levels based on findings from a conventional hearing screening. The assessment protocol included the following measures and instruments: Kaufman Brief Intelligence Test-2 (Kaufman and Kaufman 2004; nonverbal and verbal IQ), Nonword Repetition Task (NRT; Dollaghan and Campbell 1998), Syllable Repetition Task (SRT; Shriberg et al. 2009), Goldman-Fristoe Test of Articulation-2 (Goldman and Fristoe 2000; speech), Clinical Evaluation of Language Fundamentals-Preschool-2 (Wiig et al. 2004; language), Clinical Evaluation of Language Fundamentals-4 Screening Test (Semel et al. 2004; language), Woodcock–Johnson Tests of Achievement, 3rd edition (Woodcock et al. 2001; reading, spelling, and writing), and questionnaires for parent-reporting or self-reporting medical and special educational histories and concerns. One individual had a composite IQ <75, but performance on the NWR tasks was unimpaired. To maximize genetic informativeness, this individual’s NWR scores were retained for the analysis. All other individuals had an IQ between 84 and 126.

### Phenotype

The NRT is a NWR task that consists of 16 nonwords. To reduce the articulatory burden, the NRT does not contain the most phonetically complex consonants (the “late-8” consonants; Shriberg 1993; Dollaghan and Campbell 1998). Nonwords ranged in length from one to four syllables (four each) with the shortest nonwords presented first and the longest last. Each repeated consonant and vowel/diphthong (totaling 20 different phonemes) was later transcribed as correct or incorrect by two research speech pathologists. NRT scores were calculated by dividing the total phonemes correctly repeated by the total phoneme targets. Ratios were then converted to age–sex standardized scores for downstream analyses using a reference database of 200 typical speakers, ages 3–80 years (Potter et al. 2012; Scheer-Cohen et al. 2013) that included descriptive statistics for NRT and SRT scores.

The SRT is another NWR task comprised of 18 nonwords that include only four of the “early-8” consonants (/b/,/d/,/m/, and/n/) and the vowel/ɑ/(Shriberg 1993). This NWR task was designed to accommodate individuals who have incomplete phonetic inventories and/or articulatory impairments. Items range in length from two to four syllables with the shortest presented first and the longest last. The consonant responses to each recorded syllable were transcribed as correct or incorrect. The number of correctly repeated consonants was divided by the total number of target consonants. The ratio was then converted to a standard score using the reference database.

Studies of speech-language disorders using the NRT have supported its validity and reliability (e.g., Archibald and Gathercole 2006; Moore et al. 2010), including a reference sample of 95 children with typical speech and 63 children with speech delay, described in a technical report on the NRT and SRT (Shriberg and Lohmeier 2008). Findings from this reference sample include psychometric data supporting the distributional characteristics of scores for parametric statistical analyses, and analyses supporting the construct validity, concurrent validity, interjudge transcription reliability, and internal reliabilities of both tasks.

In the present study, point-to-point percentage of agreement estimates ranged from 75.6 to 88 % across nonword task and phoneme class; other validity and reliability estimates were generally in the 0.70–0.85 range. The Pearson *r* coefficient between standardized scores on the two nonword tasks in the present data was 0.66, consistent with the coefficient of 0.73 reported in Shriberg and Lohmeier (2008). Thus, consistent with discussion elsewhere, there is only moderate collinearity between the two measures of NWR (Shriberg et al. 2009).

Sensitivity and specificity for identifying speech and language disorders using the SRT were further supported by a second reference sample of 550 speakers, including speakers with typical speech and typical language, speech delay and typical language, language impairment and typical speech, and speech delay and language impairment (Lohmeier and Shriberg 2011). Additional construct validity support for the SRT was presented in Shriberg et al. (2009), followed by a series describing SRT procedures to explicate encoding, memorial, and transcoding processes underlying performance on nonword imitation tasks (Shriberg et al. 2012).

Because there is no battery of speech, language, reading, spelling, and writing tests appropriate for the lifespan ages of the present extended family, we used standardized scores from either the NRT or SRT to assign a categorical phenotype. Verbal trait impaired (Verbal Trait+) was defined as performing greater than one standard deviation below the mean on either the NRT or SRT. Preliminary studies indicated that a cutoff below one standard deviation on either the NRT or the SRT was maximally sensitive and specific to subjects with only mild, subclinical difficulty in one or more of the five verbal traits based on parent- and self-reported histories of children and adults. Of the 41.9 % of participants in the present study who met the nonword criteria for a verbal trait disorder (see Table 1), 19.2 % met criteria on the NRT only, 23.1 % met criteria on the SRT only, and 57.7 % met criteria on both nonword tasks.

**Table 1:** Percentages^a^ of affected (Verbal Trait+) and not affected (Verbal Trait−) participants in an extended family of 62 members and tests of two proportions results for each variable. Participant age was divided into four lifespan cohorts

Variable	Total *n*	Verbal trait+ (VT+)		Verbal trait− (VT−)		Tests of two proportions^b^			
		*n*	%	*n*	%	*Z*	*p*	Confidence interval	Sig.^c^
Participants	62	26	41.9	36	58.1	−1.82	0.069	−0.335, 0.012	
Gender									
Female	35	11	31.4	24	68.6	−3.35	0.001	−0.589, −0.154	*
Male	27	15	55.6	12	44.4	0.82	0.411	−0.154, 0.376	
Age									
Preschool (3–5)	3	1	33.3	2	66.7	−0.87	0.386	−1.000, 0.421	
School age (6–18)	21	7	33.3	14	66.7	−2.29	0.022	−0.618, −0.048	*
Adult (19–64)	30	14	46.7	16	53.3	−0.52	0.605	−0.319, 0.186	
Senior (65–84)	8	4	50.0	4	50.0	0.00	1.000	−0.490, 0.490	
Verbal trait history									
Speech	15	11	73.3	4	26.7	2.89	0.004	0.150, 0.783	*
Language	17	8	47.1	9	52.9	−0.34	0.731	−0.394, 0.277	
Reading	24	13	54.2	11	45.8	0.58	0.562	−0.199, 0.365	
Spelling	18	10	55.6	8	44.4	0.67	0.502	−0.214, 0.436	
Writing	3	1	33.3	2	66.7	−0.87	0.386	−1.000, 0.421	
Participants scoring more than one SD below the mean in one or more verbal trait domains	37	19	73.1	18	50.0	1.92	0.055	−0.005, 0.469	

^a^The row-wise percentages use the Total *n* in the second column as the denominator. The denominators for each percentage in the last row are 26 and 36, respectively

^b^Minitab 17 Statistical Software (2010). [Computer software]. State College, PA: Minitab, Inc. (www.minitab.com)

^c^ * *p* < 0.05

### Participants

Tables 1, 2 describe demographic and phenotype variables for participants meeting NWR task criteria for affected (Verbal Trait+; VT+) and not affected (Verbal Trait−; VT−), including tests for significant differences between the proportions of each classification. The difference in the percentages of VT+ (41.9 %) compared to VT− (58.1 %) participants was non-significant (*Z* = −1.82). Significantly fewer females met criteria for VT+ (31.4 %) than VT− (68.6 %; *Z* = −3.35), but the proportion of males who met criteria for VT+ (55.6 %) compared to the proportion who met criteria for VT− (44.4 %) was non-significant (*Z* = 0.82). Among the four age groups, the only age group within which affection status differed significantly was the school-aged participants, who had a significantly lower percentage of participants who met criteria for VT+ (33.3 %) than VT− (66.7 %; *Z* = −2.29).

**Table 2:** Descriptives for VT+ and VT− individuals in the family across the syllable repetition and nonword repetition tasks

	Verbal trait+ (VT+)	Verbal trait− (VT−)
SRT		
Mean (SD)	−3.01 (3)	0.27 (0.7)
Skewness	−0.96	0.25
Kurtosis	−0.05	0.53
NRT		
Mean (SD)	−2.07 (1.67)	0.42 (0.86)
Skewness	−0.22	0.04
Kurtosis	−0.16	−0.77

Last, Verbal Trait History for problems in verbal trait domains of speech, language, reading, spelling, and/or writing were determined by test scores in any of the relevant domains lower than one standard deviation below standardized means, or any self- or parent-reported difficulty in any of the five domains (Supplemental Table 1). Of the Verbal Trait History variables in Table 1, only one verbal trait domain was associated with significant between group proportions. A significantly greater percentage of participants with test scores, self-reported, or parental-reported histories of speech disorders met the nonword criterion for VT+ (73.3 %) compared to the percentage who met criterion for VT− (26.7 %; *Z* = 2.89). Using conventional criteria for statistical significance, the percentage of VT+ participants who had at least one test score or questionnaire entry indicating a concern with any one of the five verbal traits (73.1 %) was not significantly larger than the percentage of VT− participants with such histories (50.0 %; *Z* = 1.92; *p* = 0.055; CI −0.005, 0.469).

### DNA collection and genotyping

DNA was extracted from whole blood using the Gentra Puregene Blood Kit (Qiagen) at the University of Nebraska Medical Center. Genotyping across 551,839 single nucleotide polymorphism (SNP) markers was performed using the Illumina Infinium HumanCoreExome-24-v.1 at the Yale Center for Genome Analysis (Orange, CT). Genotypes were called using Illumina GenomeStudio with a total of 547,644 (99.24 %) passing quality control (QC). One individual failed QC due to low genotyping call rate and was excluded from the analysis.

### PBAP: marker sub-selection, pedigree structure validation, and IBD computation for linkage analysis

Reference map files for the HumanCoreExome dense marker panel were obtained from the Rutgers maps (Matise et al. 2007), with integrated linkage-physical maps in sex averaged Haldane genetic distances (cM). Reference genotype data for Europeans were extracted from the main European (EUR) population data from the 1000 genomes project (The 1000 Genomes Project Consortium 2010) to determine linkage disequilibrium (LD) and minor allele frequencies (MAF) between markers for marker sub-selection.

We used the pedigree based analysis pipeline (PBAP) to sub-select genetic markers for pedigree quality control (QC) and for interfacing with MORGAN (Thompson 2011) to calculate inheritance vectors (IV) used for linkage analysis (Nato et al. 2015). Use of MORGAN allowed multipoint analysis on the complete pedigree. Generation of genome-wide SNP marker sub-panels from the dense marker panel was conducted to (1) reduce LD between markers and minimize type 1 error, (2) reduce computational time (while maximizing genotypic informativeness within the pedigree), and (3) perform QC on pedigree structure (i.e., parent–offspring swaps). PBAP marker sub-selection and pedigree structure validation are described in detail elsewhere (Nato et al. 2015). Briefly, three non-overlapping marker sub-panels from the original dense marker panel (Illumina HumanCoreExome-24-v.1) were generated based on the following criteria: (1) maximum LD (*r* ^2^) threshold equal to 0.04 in the EUR reference population; (2) MAF 0.2–0.5 in the EUR reference population; (3) non-monomorphic marker within the pedigree;( 4) minimum intermarker distance of 0.5 cM; and (5) restricted to the 22 autosomes. A separate marker sub-panel was generated for pedigree structure validation using similar criteria as above except maximum LD threshold was equal to 0.25 and MAF from 0.3 to 0.5 in the EUR reference population. For genome-wide linkage analysis (excluding sex chromosomes), a total of 5448, 5493, and 5498 markers for sub-panels 1, 2, and 3, respectively, were created. A sub-panel of 5454 genome-wide markers was created for pedigree structure validation.

QC for appropriate parent–offspring relationships within the larger pedigree was assessed by comparing expected kinship coefficients (based on pedigree structure) and estimated coefficients computed by maximizing the likelihood from available genotype data across the 5454 marker sub-panel (Choi et al. 2009). Individual relationship pairs were flagged if the estimated kinship coefficient fell outside a 99.5 % confidence interval from expected. No sample swaps or incorrect parent–offspring relationships were observed within the larger pedigree.

From each marker sub-panel created for linkage analysis, PBAP prepared data files to generate IVs that described the flow of genetic information through a pedigree for an individual using gl_auto in the MORGAN suite of programs (Thompson 2011). The gl_auto program uses a combination of exact and Markov Chain Monte Carlo (MCMC) based estimations to sample IVs for each individual. For the current analysis, IVs were sampled for each marker subpanel using the following parameters: 15,000 MCMC burn-in iterations, sampling by scan and 100,000 MCMC iterations with progress checked every 20,000 iterations (L-Sampler = 0.2), saving 2000 realizations for IV sampling. Sampled IVs were then converted to Sequential Oligonucleotide Linkage Analysis Routines (SOLAR) (Almasy and Blangero 1998) compatible multipoint identity-by-descent (MIBD) matrices using custom scripts written by the Wijsman lab, and imported into SOLAR for downstream linkage analysis.

### Statistical analysis

All statistical analyses were conducted using the SOLAR software package (version 7.3.9; Almasy and Blangero 1998). SOLAR utilizes a maximum likelihood variance decomposition approach to estimate the influence of genetic and environmental effects on a phenotypic trait by modeling the covariance among family members relative to genetic kinship (identity by descent). A liability threshold model was used to handle discrete traits under the assumption that the affection status of an individual was determined by their underlying genetic risk exceeding a certain threshold for the phenotype (i.e., VT+; Duggirala et al. 1997). Using maximum likelihood techniques, initial models were screened for the covariate effects of age, age^2^, sex, age × sex, age^2^ × sex, and IQ. After covariate screening non-significant covariates (*p* > 0.1) were removed from the final model. In addition, a variance component for household random effects that further controlled for shared environment among nuclear families within the larger pedigree was included. The final model representing the log likelihood when the additive genetic variance was equal to 0 (no linkage elements) and covarying for household and age*sex effects, was used as the null model for hypothesis testing during linkage analysis.

Genome-wide multipoint variance component linkage analyses were conducted to examine linkage between VT+ and MIBDs. Multipoint linkage analysis considers recombination along a chromosome to determine the probability that a trait locus is located within a genomic region. Maximum likelihood estimates for linkage were calculated at approximately 0.5 cM intervals across the 22 autosomes and compared against the null model (no linkage) using a likelihood ratio test (*df* = 1).

Empirical *p* values were computed using a simulation that generated a distribution of LOD scores under a null model of no linkage. 1,000,000 simulations were conducted, each generating a random informative marker that was tested for linkage with VT+ status. This distribution of observed LOD scores in the simulation was then used to determine the empirical *p* value of the experimentally observed LOD scores.

Haplotypes were assigned using MERLIN (Abecasis et al. 2002). The large pedigree exceeded the bit limit MERLIN could handle, thus the family was split into six smaller subpedigrees for haplotype assignment and then manually reconfigured to confirm consistency of haplotypes called across the lineages. Five of the six subpedigrees were assigned based on distinct sublineages that originated from Generation II, and included all individuals within the last four generations (Generations III–VI) of the family. The sixth subpedigree consisted of all individuals in the first two generations (Generations I and II) and select individuals in Generations III and IV to confirm the transmission of the haplotypes observed in the aforementioned five subpedigrees.

## Results

**Fig. 1:**
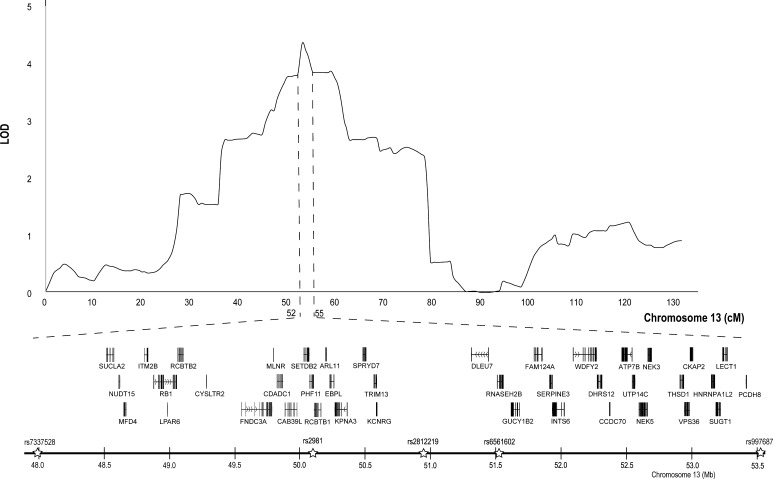
Multipoint linkage results conditioned on impaired NWR at chromosome 13. Genes and associated SNPs under the highest linkage peak of LOD = 4.35, between 52 and 55 cM spanning physical positions 48–53.5 Mb on reference genome assembly build GRCh37/hg19

Genome-wide multipoint linkage analyses for VT+ revealed a peak LOD score of 4.35 (empirical *p* value <1 × 10^−6^) between 52 and 55 cM on chromosome 13q14.2–q14.3 (Fig. 1 and Supplemental Table 2) with marker subpanel 1. This region spans base pair positions 48–53.5 Mb across 5 linkage (SNP) markers on chromosome 13, encoding a total of 41 genes (build GRCh37/hg19). To determine whether the linkage signal was due to an effect of pseudorandom marker sub sampling, multipoint analyses on chromosome 13 were conducted again using non-overlapping marker subpanels 2 and 3, which were generated at the same time as marker subpanel 1 in PBAP. Findings were recapitulated with peak LOD scores of 4.24 and 3.96 using marker subpanels 2 and 3, respectively, again spanning the same region of chromosome 13q14.2–14.3 (Supplemental Table 3). Of the 41 genes within base pair positions 48–53.5 Mb, 26 genes show moderate expression in the developing brain (BrainSpan 2011), but only 8 have known neurological or cognitive function (Carrozzo et al. 2007; de Bie et al. 2007; Dening et al. 1989; Elpeleg et al. 2005; Hilschmann et al. 2002; Jaberi et al. 2013; Kind et al. 2014; La Piana et al. 2016; Maas et al. 2015; Morris et al. 2012; Ocklenburg et al. 2015; Ostergaard et al. 2007; Rice et al. 2007; Spiechowicz et al. 2006; Vidal et al. 1999, 2000; Wei and Hemmings 2005; Xu et al. 2010; Yamagata et al. 1999; Yasuda et al. 2007; Zhang et al. 2006; Supplemental Table 4). When considering LOD scores greater than 3 (empirical *p* value <0.0002), the linkage signal expands to 46–61 cM across 22 linkage markers spanning a 23.2 Mb region on chromosome 13q14.11-21.32, encompassing approximately 77 genes.

**Fig. 2:**
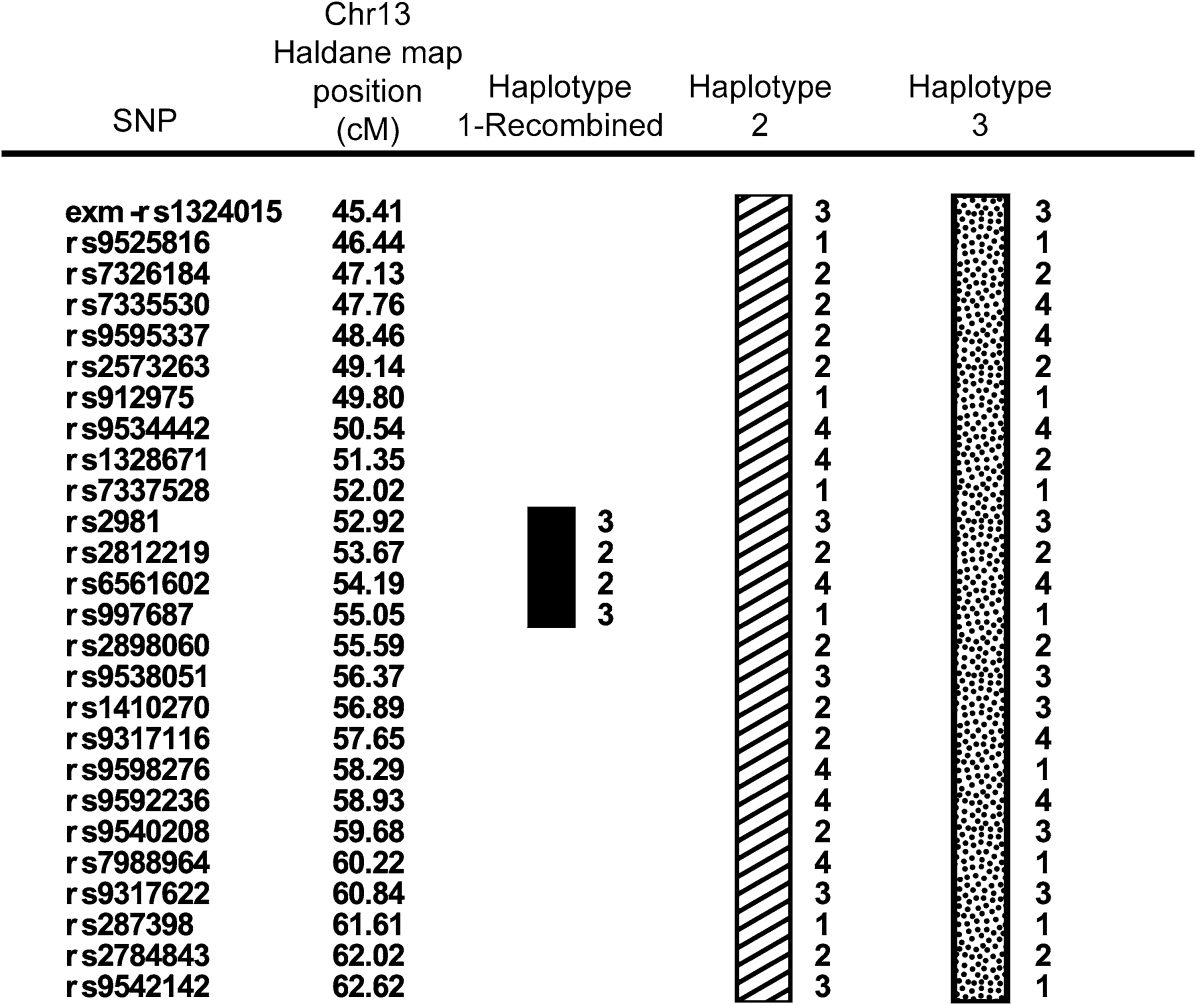
Haplotypes spanning genomic location 45–62 cM on chromosome 13 segregating with Verbal Trait+ (affected) status in the family. The centromeric and telomeric boundaries of Haplotype 1-Recombined are defined by a recombinatorial events within individual 3 (Fig. 3). No recombinatorial events in the family offer clear centromeric and telomeric boundaries for Haplotypes 2 and 3

**Fig. 3:**
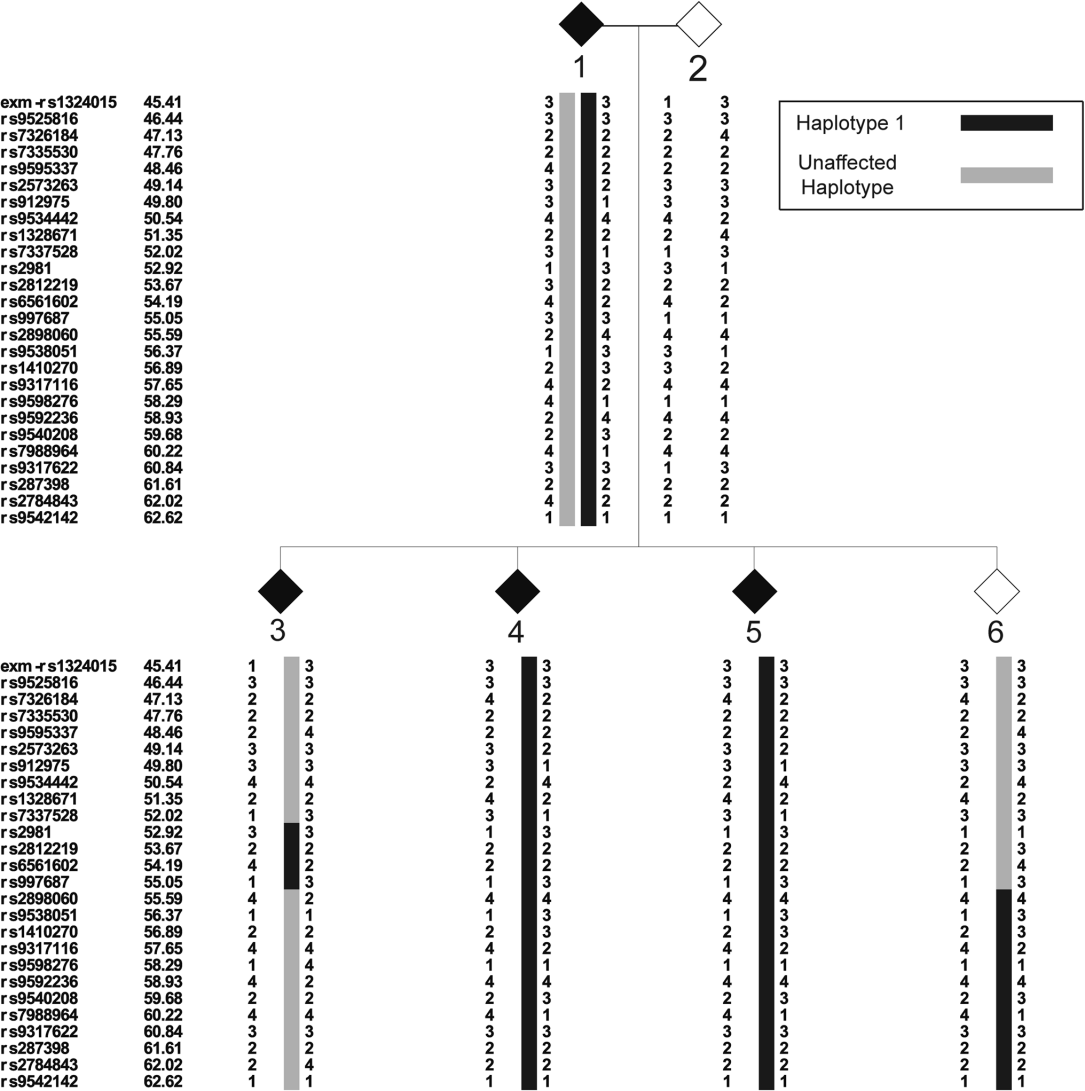
Haplotype assignments spanning genomic location 45–62 cM on chromosome 13. The pedigree depicted is truncated to reflect the recombinatorial event observed in affected individual 3 that outlines the centromeric and telomeric border of the linkage signal spanning 52–55 cM. Affected individuals are black diamonds, while unaffected individuals are gray diamonds

Estimation of haplotypes in the extended family provides evidence of at least 3 distinct haplotypes on chromosome 13 segregating with VT+ (Fig. 2). A recombination in Haplotype 1 (Haplotype 1-recombined) in affected individual 3 between SNPs rs7337528 and rs2981 defines the centromeric boundary of LOD = 4.35 at 52 cM (Figs. 2, 3). This particular segment of Haplotype 1, defined by alleles shared IBD at rs2981, rs2812219, rs6561602, and rs997687 (52.92–55.06 cM), segregates with the phenotype in the lineage originated by a founder in the oldest generation. For the remaining information, data are presented without a conventional haplotype pedigree graphic to preserve anonymity of the family. Haplotype 2 originates from a married-in founder in Generation II and segregates to two siblings in Generation III, four descendants in Generation IV and four descendants in Generation V. Seven of ten descendants with Haplotype 2 are VT+. Last, Haplotype 3 originates from a married-in founder in Generation III, segregates to two siblings in generation IV, and four descendants in Generation V. Six of seven pedigree members with Haplotype 3 are VT+. There are no recombinations within Haplotypes 2 or 3 that would define the broader shoulders of the linkage signal from 45 to 62 cM (Fig. 3). Overall, the presence of at least three distinct haplotypes that cosegregate with VT+—including married-in Haplotypes 2 and 3—implicate multiple contributing variants segregating through this family.

Other suggestive linkage signals (LOD >1.5; empirical *p* value <0.01) were observed on chromosome 2q37.1, 4q12–13.2, 4q25, 7q22.3–31.2, 8q24.3, and 12p13.33 (Supplemental Table 2). Most notably, the linkage peak spanning 7q22.3–q31.2 has a max LOD = 2.06 and contains the gene FOXP2—a causal gene for Childhood Apraxia of Speech (Fisher et al. 1998; Lai et al. 2001).

## Discussion

The present study identified a linkage signal spanning chromosome 13q14–q21 using a categorical phenotype (VT+ or VT−) derived from performance on NWR in an extended pedigree with a history of verbal trait disorders. This region encompasses SLI3 on chromosome 13q21, a SLI locus previously identified by Bartlett et al. (2002), using a family-based linkage analysis in five Canadian families of Celtic ancestry with a history of specific language impairment (SLI). Their analysis was conditioned on a categorical reading-IQ discrepancy phenotype (nonword reading score at least one standard deviation below performance IQ), which they replicated in a larger independent US sample using the same phenotype (Bartlett et al. 2004). This region has also been implicated in autism spectrum disorder (ASD), a neurodevelopmental disorder with a core language component in combination with other core abnormalities in social and repetitive behaviors. A linkage signal at chromosome 13q21 was observed with a language delay phenotype in the Collaborative Linkage Study of Autism (CLSA; Bradford et al. 2001). Furthermore, deletions at 13q12 through 13q21 have been reported in three subjects with ASD and poor receptive and expressive vocabulary (but normal speech), and in a subject with ASD with auditory processing deficits (Mitter et al. 2011; Smith et al. 2002; Steele et al. 2001). These deleted segments partially overlap the linkage peak in our present study, where LOD >1.5 (Supplemental Tables 2 and 3). The convergence of these findings associated with 13q14–q21 with related language phenotypes that underlie verbal trait disorders provides compelling support for this locus.

Within this pedigree, there are at least three distinct haplotypes segregating with VT+, of which, only Haplotype 1 originated with a founder in the oldest generation—the other two are more recently married into, consistent with assortative mating. Within the EUR reference population of 1000 genomes project, Haplotype 1-Recombined (Fig. 2) is common with a frequency of 0.046. In a clinical context, verbal trait disorders such as developmental dyslexia and specific language impairment (SLI) have a high prevalence in the United States. The prevalence of developmental dyslexia is 7 % in the general population, and the prevalence of specific language impairment (SLI) is 5–8 % among preschool children (Peterson and Pennington 2015; Tomblin et al. 1997). Different haplotypes segregating within the family could indicate a single gene with different causal variants segregating within the family. It is also possible that different genes at the same locus, or at different loci, are mediating NWR performance. Further fine mapping and sequencing of the region is necessary to disentangle these possibilities and elucidate the potential variants driving the signal observed in the present study.

Underneath the peak linkage signal spanning the 52–55 cM region of chromosome 13 with LOD >4, there are interesting gene candidates with known function in neuropsychiatric disorders and neurodevelopment. *ITM2B* encodes a transmembrane protein that helps to inhibit the accumulation of beta-amyloid, but mutations have been implicated in Familial British Dementia and Familial Danish Dementia with similar pathology to Alzheimer disease (Vidal et al. 1999, 2000). *Setdb2*, the zebrafish ortholog of *SETDB2*, is known to regulate left–right asymmetry in the zebrafish central nervous system (Xu et al. 2010). Human epidemiological research has also associated *SETDB2* with handedness (left versus right preference) with specific variants linked to reduction in laterality (Ocklenburg et al. 2015). This provides an interesting parallel to previous evidence that suggests language and reading disability are linked to atypical cerebral laterality (asymmetry) since language-related behavior is typically left lateralized (Leonard and Eckert 2008; Scerri et al. 2011). However, it is important to note that *SETDB2* has not yet been directly implicated in reading or language disability. *ATP7B* is a known gene associated with Wilson disease, which is a disorder characterized by the deposition of copper in the liver, brain, and other tissues, leading to neurological and cognitive deterioration including memory loss, tremors, and emotional changes (de Bie et al. 2007; Dening Tr 1989). *PCDH8* is part of the protocadherin family of CNS-specific cell adhesion molecules that plays a role in the development of neural circuitry (Hilschmann et al. 2002; Yamagata et al. 1999). Interestingly, the rat ortholog of *PCDH8*, *Arcadlin*, has been implicated in synaptic function and is dynamically expressed upon activation of hippocampal circuitry—a neural network necessary for learning and memory (Yasuda et al. 2007).

By examining the extended linkage peak spanning 45–62 cM with LOD scores >3, we identified another three genes in the protocadherin family located telemetric to *PCDH8*—*PCDH17*, *PCDH20*, and *PCDH*—each of which encodes cell–cell adhesion molecules that are primarily expressed in the brain (Kim et al. 2010). Variation in *PCDH9* has been linked to ASD and SLI, while *PCDH17* is highly expressed in the prefrontal and anterior regions of the temporal cortex and subcortical structures such as the thalamus, ventral striatum, and anterior cingulate—an expression pattern highlighting an overlap with corticostriatothalamic circuitry critical for higher order cognitive function and language development (Abrahams et al. 2007; Marshall et al. 2008).

Our linkage findings on chromosome 13q do not correspond to other genome-wide linkage scans conditioned on NWR. A family-based linkage analysis conducted by the SLI consortium localized to chromosome 16q with further fine mapping identifying *CMIP* and *ATP2C2* as potential gene candidates mediating NWR in their sample (SLI Consortium 2002, 2004; Newbury et al. 2009). Another study performed by Brkanac et al. (2008), observed linkage signals on chromosomes 4p12, 12p, and 17q in families with a history of dyslexia. Discrepancies in genomic regions associated with NWR, in part, may be due to differences in the particular NWR test used to evaluate respective subjects. Although the measures used in these studies do ostensibly assess NWR, there are differences in each that may more heavily tap into different combinations of underlying cognitive and/or behavioral abilities, such as phonological working memory, long-term lexical knowledge, and articulatory difficulty with nonsense words (Estes et al. 2007; Gathercole 1995). Ascertainment differences and differences in age ranges between the present study and others could also contribute to the observed discrepancies. The SLI consortium used a family-based linkage analysis examining 186 nuclear families affected with SLI (SLI Consortium 2002, 2004). Brkanac et al. (2008), used a family-based design examining 144 families with a history of dyslexia, whereas the present study examined one extended family with verbal trait disorder that could be derived from one or more rare variants. The small number of subjects in each of these studies would significantly limit the power to detect rare and uncommon variants. Ultimately, these findings may also reflect locus heterogeneity and highlight different molecular and biological mechanisms associated with NWR.

Due to the complex inheritance pattern of impaired NWR performance, a nonparametric analysis using variance components was used so that pre-specified values for parameters defining the genetic model would not be required (Bailey-Wilson 2004). This is in contrast to a parametric analysis that requires the specification of a genetic model, with the concern that a poorly specified model could lead to suboptimal results. An advantage to using a variance components approach is that it tends to be more powerful relative to other trait mapping methods (Kleensang et al. 2010). However, a limitation is that variance components provides poorer localization of the trait locus compared to a parametric analysis, and generally requires additional fine mapping to isolate the region (Amos and de Andrade 2001; Williams et al. 1997). An additional limitation is that we used a composite variable across two different NWR tests—performing more than one standard deviation below the age–sex standardized mean on either the NRT or SRT—to derive VT+ or − status. Although both measures evaluate NWR, the individual test items differ. As described previously, the SRT focuses on the repetition of syllables that comprise only four “early-8” consonants and can be used to examine NWR ability in young children and speakers of any age with limited phonetic inventories or speech sound disorder (Shriberg et al. 2009). In comparison, although the NRT was designed to exclude late developing English consonants (the ‘late-8’), younger children and speakers of any age with speech sound disorder can have articulation errors repeating the 9 different vowels and diphthongs, and 11 different consonants in the nonsense words, thus confounding test performance and reducing transcription reliability (Shriberg et al. 2009). Because individuals tested in the extended pedigree ranged in age from 3 to 95 years old, it would not be optimal to test all individuals on only the NRT or SRT, as they may differ in their sensitivity to persistent types and levels of NWR deficits across the lifespan.

In conclusion, we found a statistically significant genome-wide multipoint linkage signal on chromosome 13q14–q21 using a NWR phenotype in an extended pedigree with a family history of verbal trait disorder. We hypothesize that the region of 13q14–q21 is a susceptibility locus for verbal trait disorders, but additional work must be conducted to (1) identify the gene(s) in this region contributing to the linkage signals observed in the present study and others that have been identified this same region, and (2) elucidate the complex genetic and environmental interactions that may increase susceptibility.

## Electronic supplementary material

Below is the link to the electronic supplementary material.

Supplementary material 1 (DOCX 13 kb)

Supplementary material 2 (DOCX 20 kb)

Supplementary material 3 (DOCX 18 kb)

Supplementary material 4 (DOCX 30 kb)
