# Supplementary material for: Multipoint genome-wide linkage scan for nonword repetition in a multigenerational family further supports chromosome 13q as a locus for verbal trait disorders
Source: Hum Genet. 2016 Aug 17;135(12):1329–41. doi: 10.1007/s00439-016-1717-z (PMC5065602; doi:10.1007/s00439-016-1717-z)
Supplement: Supplementary file 1 — Supplementary material 1 (DOCX 13 kb) [file 439_2016_1717_MOESM1_ESM.docx]

**Supplemental Table 1.** Assessments to define Verbal Trait History and relevant verbal trait domains

| **Neurocognitive Assessment** | **Verbal Trait Domain** |
| --- | --- |
|  |  |
| Goldman-Fristoe Test of Articulation-2 | Speech |
|  |  |
| Clinical Evaluation of Language Fundamentals-Preschool-2 | Language |
|  |  |
| Clinical Evaluation of Language Fundamentals-4 Screening Test | Language |
|  |  |
| Woodcock–Johnson Tests of Achievement, 3rd edition | Reading, Spelling, and Writing |
|  |  |
| Parental Questionnaire and Self-report | Speech, Language, Reading, Spelling, and Writing |
