# Supplementary material for: Multipoint genome-wide linkage scan for nonword repetition in a multigenerational family further supports chromosome 13q as a locus for verbal trait disorders
Source: Hum Genet. 2016 Aug 17;135(12):1329–41. doi: 10.1007/s00439-016-1717-z (PMC5065602; doi:10.1007/s00439-016-1717-z)
Supplement: Supplementary file 2 — Supplementary material 2 (DOCX 20 kb) [file 439_2016_1717_MOESM2_ESM.docx]

| **Supplemental Table 2.** Multipoint linkage results from chromosomes 2, 4, 7, 8, 12, and 13 on marker subpanel 1. | | |
| --- | --- | --- |
|  | | |
| Chromosome | cM | LOD |
| 2 | 223.671 | 1.0275 |
| 2 | 224.4823 | 1.1251 |
| 2 | 225.1164 | 1.1861 |
| 2 | 225.6522 | 1.1074 |
| 2 | 226.3403 | 1.1876 |
| 2 | 226.8875 | 1.2522 |
| 2 | 227.472 | 1.2993 |
| 2 | 228.07 | 1.2903 |
| 2 | 228.6086 | 1.292 |
| 2 | 229.5753 | 1.2884 |
| 2 | 230.0798 | 1.2846 |
| 2 | 230.7841 | 1.2794 |
| 2 | 231.611 | 1.2739 |
| 2 | 232.2687 | 1.2729 |
| 2 | 232.7785 | 1.2748 |
| 2 | 233.5808 | 1.2789 |
| 2 | 233.9876 | 1.228 |
| 2 | 234.753 | 1.227 |
| 2 | 235.1732 | 1.2466 |
| 2 | 235.9096 | 1.3361 |
| 2 | 236.6441 | 1.3673 |
| 2 | 237.2624 | 2.1079 |
| 2 | 237.8529 | 2.0993 |
| 2 | 238.7296 | 2.0889 |
| 2 | 239.2553 | 2.0802 |
| 2 | 239.8902 | 2.069 |
| 2 | 240.5662 | 2.0698 |
| 2 | 241.1428 | 2.0689 |
| 2 | 241.6842 | 2.0688 |
| 2 | 242.1565 | 2.0685 |
| 2 | 242.5602 | 2.0638 |
| 2 | 243.3254 | 2.0628 |
| 2 | 243.826 | 2.0667 |
| 2 | 244.231 | 2.0113 |
| 2 | 244.7085 | 2.0575 |
| 2 | 245.248 | 2.0586 |
| 2 | 245.8131 | 1.6309 |
| 2 | 246.5091 | 1.5487 |
| 2 | 247.1207 | 1.4566 |
| 2 | 247.8713 | 1.2743 |
| 2 | 248.4146 | 1.1269 |
| 2 | 248.9963 | 1.1134 |
| 2 | 249.8048 | 1.1594 |
| 2 | 250.7002 | 1.1508 |
| 2 | 251.2953 | 1.088 |

| Chromosome | cM | LOD |
| --- | --- | --- |
| 4 | 64.147754 | 0.7264 |
| 4 | 64.880503 | 1.1258 |
| 4 | 65.565 | 1.4608 |
| 4 | 66.194569 | 1.4638 |
| 4 | 66.75312 | 1.5755 |
| 4 | 67.163045 | 1.6357 |
| 4 | 68.02766 | 1.7337 |
| 4 | 68.821642 | 1.8105 |
| 4 | 69.455045 | 1.8663 |
| 4 | 69.893434 | 1.8963 |
| 4 | 70.455139 | 1.9303 |
| 4 | 71.119706 | 1.9674 |
| 4 | 71.527252 | 1.9212 |
| 4 | 72.290359 | 1.857 |
| 4 | 72.904022 | 1.8363 |
| 4 | 73.69587 | 1.8915 |
| 4 | 74.247671 | 1.8783 |
| 4 | 74.801488 | 1.7668 |
| 4 | 75.369861 | 1.6185 |
| 4 | 76.024848 | 1.6157 |
| 4 | 76.861434 | 1.6123 |
| 4 | 77.376309 | 1.6112 |
| 4 | 77.938844 | 1.611 |
| 4 | 78.516713 | 1.612 |
| 4 | 79.210507 | 1.6109 |
| 4 | 79.878985 | 1.6101 |
| 4 | 80.513323 | 1.5206 |
| 4 | 81.143237 | 1.3745 |
| 4 | 81.821747 | 1.1237 |
| 4 | 82.388019 | 1.0736 |
| 4 | 82.936132 | 1.0168 |
| 4 | 83.856721 | 1.0432 |
| 4 | 84.670331 | 1.0684 |
| 4 | 85.241665 | 1.089 |
| 4 | 85.882698 | 1.0941 |
| 4 | 86.497431 | 1.0928 |
| 4 | 87.289024 | 1.1582 |
| 4 | 87.80615 | 1.106 |
| 4 | 88.31538 | 0.9791 |

| Chromosome | cM | LOD |
| --- | --- | --- |
| 4 | 107.208683 | 0.9468 |
| 4 | 108.007685 | 1.0167 |
| 4 | 108.655005 | 1.0587 |
| 4 | 109.347305 | 1.079 |
| 4 | 109.987311 | 1.0809 |
| 4 | 110.837404 | 1.0852 |
| 4 | 111.809003 | 1.0683 |
| 4 | 112.597594 | 1.0558 |
| 4 | 113.371974 | 1.0432 |
| 4 | 114.014833 | 1.0231 |
| 4 | 114.605538 | 0.9916 |
| 4 | 115.248596 | 1.0779 |
| 4 | 115.899622 | 1.3073 |
| 4 | 116.878298 | 1.228 |
| 4 | 117.411305 | 1.647 |
| 4 | 117.958463 | 1.7599 |
| 4 | 118.82647 | 1.7507 |
| 4 | 119.4895 | 1.7434 |
| 4 | 120.024811 | 1.7356 |
| 4 | 120.431178 | 1.7305 |
| 4 | 121.036497 | 1.6128 |
| 4 | 121.71742 | 1.5593 |
| 4 | 122.414954 | 1.387 |
| 4 | 123.185011 | 1.15 |
| 4 | 123.810425 | 1.0502 |
| 4 | 124.360478 | 0.974 |

| Chromosome | cM | LOD |
| --- | --- | --- |
| 7 | 108.8217 | 0.8912 |
| 7 | 109.5337 | 1.2652 |
| 7 | 110.3768 | 1.2664 |
| 7 | 110.9381 | 1.2651 |
| 7 | 111.7247 | 1.2674 |
| 7 | 112.2891 | 1.3681 |
| 7 | 112.9313 | 1.4327 |
| 7 | 113.9111 | 1.6268 |
| 7 | 114.5724 | 1.7471 |
| 7 | 115.373 | 1.7608 |
| 7 | 116.0666 | 1.7934 |
| 7 | 116.4691 | 1.8032 |
| 7 | 117.3786 | 1.7919 |
| 7 | 117.9251 | 1.7664 |
| 7 | 118.7162 | 1.7264 |
| 7 | 119.5354 | 1.7283 |
| 7 | 120.124 | 1.7121 |
| 7 | 120.7664 | 1.6895 |
| 7 | 121.325 | 1.6749 |
| 7 | 121.9313 | 2.0658 |
| 7 | 122.918 | 1.7943 |
| 7 | 123.6427 | 1.7026 |
| 7 | 124.1828 | 1.7136 |
| 7 | 124.8214 | 1.4902 |
| 7 | 125.6135 | 1.4717 |
| 7 | 126.345 | 1.2408 |
| 7 | 126.918 | 1.2019 |
| 7 | 127.5111 | 1.0569 |
| 7 | 128.0976 | 0.8524 |

| Chromosome | cM | LOD |
| --- | --- | --- |
| 8 | 164.5752 | 0.8591 |
| 8 | 165.1422 | 1.1318 |
| 8 | 165.775 | 1.4665 |
| 8 | 166.2818 | 1.4507 |
| 8 | 167.2129 | 1.5738 |
| 8 | 167.7357 | 1.6048 |
| 8 | 168.186 | 1.6139 |
| 8 | 168.8723 | 1.6043 |

| Chromosome | cM | LOD |
| --- | --- | --- |
| 12 | 0 | 1.8028 |
| 12 | 0.615765 | 1.8151 |
| 12 | 1.196118 | 1.8388 |
| 12 | 1.597608 | 1.8437 |
| 12 | 2.02686 | 1.8432 |
| 12 | 3.176873 | 1.9357 |
| 12 | 3.889288 | 1.84 |
| 12 | 4.475307 | 1.8818 |
| 12 | 4.886339 | 1.9105 |
| 12 | 5.680165 | 1.9675 |
| 12 | 6.196109 | 0.2888 |

| Chromosome | cM | LOD |
| --- | --- | --- |
| 13 | 26.23022 | 0.7445 |
| 13 | 27.07336 | 1.1118 |
| 13 | 27.78123 | 1.7045 |
| 13 | 28.19194 | 1.713 |
| 13 | 28.96863 | 1.7281 |
| 13 | 29.5527 | 1.7307 |
| 13 | 30.10048 | 1.7107 |
| 13 | 30.85814 | 1.6394 |
| 13 | 31.51679 | 1.54 |
| 13 | 32.19491 | 1.5605 |
| 13 | 32.69797 | 1.5458 |
| 13 | 33.09869 | 1.5379 |
| 13 | 33.57685 | 1.5378 |
| 13 | 34.05739 | 1.5383 |
| 13 | 34.59006 | 1.5387 |
| 13 | 34.99486 | 1.5413 |
| 13 | 35.60294 | 1.5473 |
| 13 | 36.26937 | 2.4418 |
| 13 | 36.9805 | 2.6644 |
| 13 | 37.55456 | 2.6602 |
| 13 | 38.44203 | 2.6603 |
| 13 | 39.18918 | 2.6745 |
| 13 | 39.79579 | 2.678 |
| 13 | 40.63899 | 2.6816 |
| 13 | 41.48371 | 2.6919 |
| 13 | 42.17615 | 2.7438 |
| 13 | 42.71829 | 2.7817 |
| 13 | 43.42938 | 2.7704 |
| 13 | 43.97258 | 2.7618 |
| 13 | 44.68412 | 2.7608 |
| 13 | 45.40926 | 2.9819 |
| 13 | 46.43887 | 3.182 |
| 13 | 47.13353 | 3.1765 |
| 13 | 47.76312 | 3.3794 |
| 13 | 48.46065 | 3.5276 |
| 13 | 49.1368 | 3.6466 |
| 13 | 49.80392 | 3.7588 |
| 13 | 50.5445 | 3.7672 |
| 13 | 51.3516 | 3.787 |
| 13 | 52.02244 | 3.8089 |
| 13 | 52.91963 | 4.3465 |
| 13 | 53.67407 | 4.2213 |
| 13 | 54.19479 | 4.1128 |
| 13 | 55.05207 | 3.8518 |
| 13 | 55.58859 | 3.8396 |
| 13 | 56.37179 | 3.8391 |
| 13 | 56.89297 | 3.8395 |
| 13 | 57.64662 | 3.8372 |
| 13 | 58.29477 | 3.8386 |
| 13 | 58.92767 | 3.8604 |
| 13 | 59.68201 | 3.7383 |
| 13 | 60.21729 | 3.639 |
| 13 | 60.84417 | 3.3955 |
| 13 | 61.61074 | 2.9893 |
| 13 | 62.01546 | 2.8772 |
| 13 | 62.62272 | 2.6693 |
| 13 | 63.33653 | 2.6694 |
| 13 | 63.94263 | 2.6692 |
| 13 | 64.83501 | 2.6692 |
| 13 | 65.72258 | 2.6689 |
| 13 | 66.39213 | 2.6982 |
| 13 | 66.80528 | 2.7023 |
| 13 | 67.53892 | 2.7102 |
| 13 | 68.34411 | 2.6837 |
| 13 | 68.93225 | 2.4758 |
| 13 | 69.49027 | 2.488 |
| 13 | 70.06988 | 2.4993 |
| 13 | 70.5867 | 2.5119 |
| 13 | 71.11389 | 2.5238 |
| 13 | 71.71778 | 2.4339 |
| 13 | 72.35017 | 2.4342 |
| 13 | 73.31449 | 2.5003 |
| 13 | 73.99659 | 2.5284 |
| 13 | 74.60718 | 2.5358 |
| 13 | 75.59195 | 2.5055 |
| 13 | 76.14643 | 2.4829 |
| 13 | 76.80976 | 2.4518 |
| 13 | 77.44617 | 2.4145 |
| 13 | 78.16813 | 2.3587 |
| 13 | 78.73897 | 1.7581 |
| 13 | 79.3794 | 0.5345 |
