# Supplementary material for: Multipoint genome-wide linkage scan for nonword repetition in a multigenerational family further supports chromosome 13q as a locus for verbal trait disorders
Source: Hum Genet. 2016 Aug 17;135(12):1329–41. doi: 10.1007/s00439-016-1717-z (PMC5065602; doi:10.1007/s00439-016-1717-z)
Supplement: Supplementary file 3 — Supplementary material 3 (DOCX 18 kb) [file 439_2016_1717_MOESM3_ESM.docx]

| **Supplemental Table 3.** Multipoint linkage results from subpanels 2 and 3 of chromosome 13. | | | |
| --- | --- | --- | --- |
|  | | | |
|  | Chromosome | cM | LOD |
| Sub-panel 2 | 13 | 26.24322 | 0.3851 |
|  | 13 | 26.83562 | 0.4886 |
|  | 13 | 27.78772 | 0.8771 |
|  | 13 | 28.47512 | 1.1674 |
|  | 13 | 29.41244 | 1.6637 |
|  | 13 | 30.10598 | 1.6453 |
|  | 13 | 30.93568 | 1.6171 |
|  | 13 | 31.4482 | 1.6254 |
|  | 13 | 32.19984 | 1.5808 |
|  | 13 | 32.70755 | 1.5775 |
|  | 13 | 33.1149 | 1.5614 |
|  | 13 | 33.57685 | 1.5588 |
|  | 13 | 34.0687 | 1.5581 |
|  | 13 | 34.59633 | 1.56 |
|  | 13 | 35.5021 | 2.4166 |
|  | 13 | 36.14348 | 2.583 |
|  | 13 | 36.73499 | 2.6532 |
|  | 13 | 37.23855 | 2.6403 |
|  | 13 | 37.78596 | 2.6251 |
|  | 13 | 38.54459 | 2.6057 |
|  | 13 | 39.37418 | 2.5912 |
|  | 13 | 40.18725 | 2.5747 |
|  | 13 | 40.74893 | 2.5556 |
|  | 13 | 41.26087 | 2.58 |
|  | 13 | 42.20656 | 2.6072 |
|  | 13 | 42.96944 | 2.7052 |
|  | 13 | 43.68868 | 2.7344 |
|  | 13 | 44.36667 | 2.7686 |
|  | 13 | 44.95596 | 2.7686 |
|  | 13 | 45.52374 | 2.9672 |
|  | 13 | 46.43948 | 3.1761 |
|  | 13 | 47.26914 | 3.2864 |
|  | 13 | 47.87592 | 3.5074 |
|  | 13 | 48.46321 | 3.6399 |
|  | 13 | 49.13842 | 3.7731 |
|  | 13 | 49.92164 | 3.9005 |
|  | 13 | 50.86315 | 3.9067 |
|  | 13 | 51.66604 | 4.1279 |
|  | 13 | 52.26235 | 4.2035 |
|  | 13 | 52.81844 | 4.2409 |
|  | 13 | 53.46894 | 4.1834 |
|  | 13 | 54.02419 | 4.1042 |
|  | 13 | 54.74002 | 3.8123 |
|  | 13 | 55.48495 | 3.836 |
|  | 13 | 56.37351 | 3.8791 |
|  | 13 | 56.89719 | 3.8606 |
|  | 13 | 57.64872 | 3.7752 |
|  | 13 | 58.29557 | 3.7604 |
|  | 13 | 58.93119 | 3.75 |
|  | 13 | 59.68389 | 3.6142 |
|  | 13 | 60.22177 | 3.4974 |
|  | 13 | 60.84656 | 2.7499 |
|  | 13 | 61.50329 | 2.5644 |
|  | 13 | 62.2841 | 2.6038 |
|  | 13 | 63.05365 | 2.6685 |
|  | 13 | 63.94789 | 2.6673 |
|  | 13 | 64.8396 | 2.6675 |
|  | 13 | 65.7779 | 2.6563 |
|  | 13 | 66.39576 | 2.6768 |
|  | 13 | 66.80528 | 2.6782 |
|  | 13 | 67.57086 | 2.6363 |
|  | 13 | 68.14546 | 2.5861 |
|  | 13 | 68.93588 | 2.5076 |
|  | 13 | 69.50553 | 2.4585 |
|  | 13 | 70.08083 | 2.4702 |
|  | 13 | 71.00113 | 2.4953 |
|  | 13 | 71.60528 | 2.4591 |
|  | 13 | 72.1945 | 2.4301 |
|  | 13 | 72.84506 | 2.4253 |
|  | 13 | 73.26516 | 2.4245 |
|  | 13 | 73.89161 | 2.4787 |
|  | 13 | 74.74335 | 2.5086 |
|  | 13 | 75.69579 | 2.5382 |
|  | 13 | 76.2868 | 2.5043 |
|  | 13 | 76.94439 | 2.4601 |
|  | 13 | 77.58334 | 2.379 |
|  | 13 | 78.27951 | 2.3553 |
|  | 13 | 78.85318 | 2.0581 |
|  | 13 | 79.60999 | 1.5056 |
|  | 13 | 80.39649 | 0.5283 |

|  | Chromosome | cM | LOD |
| --- | --- | --- | --- |
| Sub-panel 3 | 13 | 26.36487 | 0.3834 |
|  | 13 | 27.08395 | 0.488 |
|  | 13 | 28.04855 | 0.5529 |
|  | 13 | 28.47512 | 0.5762 |
|  | 13 | 29.17365 | 0.5863 |
|  | 13 | 29.70846 | 0.5979 |
|  | 13 | 30.1631 | 0.6205 |
|  | 13 | 31.05643 | 0.7981 |
|  | 13 | 31.77977 | 0.9167 |
|  | 13 | 32.30905 | 1.0014 |
|  | 13 | 33.02463 | 1.0508 |
|  | 13 | 33.76065 | 1.1263 |
|  | 13 | 34.6053 | 1.2308 |
|  | 13 | 35.37052 | 1.3925 |
|  | 13 | 36.0346 | 1.5005 |
|  | 13 | 36.73938 | 2.657 |
|  | 13 | 37.24681 | 2.6681 |
|  | 13 | 37.81319 | 2.6684 |
|  | 13 | 38.25639 | 2.6694 |
|  | 13 | 38.88933 | 2.669 |
|  | 13 | 39.50286 | 2.6704 |
|  | 13 | 40.19699 | 2.6947 |
|  | 13 | 41.12475 | 2.7571 |
|  | 13 | 41.72498 | 2.7927 |
|  | 13 | 42.14504 | 2.8128 |
|  | 13 | 42.72625 | 2.8188 |
|  | 13 | 43.31915 | 2.8017 |
|  | 13 | 44.25341 | 2.765 |
|  | 13 | 44.8405 | 2.7618 |
|  | 13 | 45.41803 | 2.9576 |
|  | 13 | 45.9289 | 3.0886 |
|  | 13 | 46.45734 | 3.1856 |
|  | 13 | 47.27183 | 3.2899 |
|  | 13 | 47.87913 | 3.2914 |
|  | 13 | 48.62692 | 3.3088 |
|  | 13 | 49.37195 | 3.6946 |
|  | 13 | 50.09406 | 3.9206 |
|  | 13 | 50.87694 | 3.9249 |
|  | 13 | 51.4419 | 3.9183 |
|  | 13 | 52.26264 | 3.8927 |
|  | 13 | 52.8355 | 3.9643 |
|  | 13 | 53.48029 | 3.9585 |
|  | 13 | 54.02752 | 3.8883 |
|  | 13 | 54.53772 | 3.8101 |
|  | 13 | 55.16395 | 3.8131 |
|  | 13 | 55.693 | 3.863 |
|  | 13 | 56.24957 | 3.8596 |
|  | 13 | 56.82556 | 3.8593 |
|  | 13 | 57.42597 | 3.8591 |
|  | 13 | 57.99919 | 3.8483 |
|  | 13 | 58.64125 | 3.7321 |
|  | 13 | 59.31845 | 3.5901 |
|  | 13 | 59.87521 | 3.4419 |
|  | 13 | 60.53604 | 3.1688 |
|  | 13 | 61.29843 | 3.0486 |
|  | 13 | 61.84769 | 2.9132 |
|  | 13 | 62.56954 | 2.6363 |
|  | 13 | 63.105 | 2.6366 |
|  | 13 | 63.82764 | 2.6373 |
|  | 13 | 64.86337 | 2.6306 |
|  | 13 | 65.60551 | 2.6315 |
|  | 13 | 66.15476 | 2.6379 |
|  | 13 | 66.58941 | 2.6483 |
|  | 13 | 67.29837 | 2.6692 |
|  | 13 | 68.04327 | 2.6527 |
|  | 13 | 68.60326 | 2.4469 |
|  | 13 | 69.12938 | 2.4471 |
|  | 13 | 70.00552 | 2.4461 |
|  | 13 | 70.72112 | 2.4643 |
|  | 13 | 71.48841 | 2.4067 |
|  | 13 | 72.2113 | 2.4299 |
|  | 13 | 72.74505 | 2.4627 |
|  | 13 | 73.43119 | 2.4888 |
|  | 13 | 74.01333 | 2.5183 |
|  | 13 | 74.76263 | 2.5326 |
|  | 13 | 75.47785 | 2.541 |
|  | 13 | 76.03196 | 2.5444 |
|  | 13 | 76.83972 | 2.2039 |
|  | 13 | 77.51953 | 1.8301 |
|  | 13 | 78.21488 | 1.3878 |
|  | 13 | 78.75227 | 1.0041 |
|  | 13 | 79.40262 | 0.531 |
