# Supplementary material for: Multipoint genome-wide linkage scan for nonword repetition in a multigenerational family further supports chromosome 13q as a locus for verbal trait disorders
Source: Hum Genet. 2016 Aug 17;135(12):1329–41. doi: 10.1007/s00439-016-1717-z (PMC5065602; doi:10.1007/s00439-016-1717-z)
Supplement: Supplementary file 4 — Supplementary material 4 (DOCX 30 kb) [file 439_2016_1717_MOESM4_ESM.docx]

**Supplemental Table 4.** Genes located in cM positions 48-53.5 Mb with associated neurological or cognitive function.

| **Gene** | **Chr** | **Bp start** | **Bp end** | **Associated neurological or cognitive function** |
| --- | --- | --- | --- | --- |
| SUCLA2 | 13 | 48516790 | 48575462 | Encephalomyopathy (Carrozzo et al. 2007; Elpeleg et al. 2005; Jaberi et al. 2013; Maas et al. 2015; Ostergaard et al. 2007) |
|  |  |  |  |  |
| ITM2B | 13 | 48807273 | 48836232 | Dementia (Vidal et al. 1999; Vidal et al. 2000) |
|  |  |  |  |  |
| SETDB2 | 13 | 50025688 | 50069139 | Left/Right asymmetry in central nervous system (Ocklenburg et al. 2015; Xu et al. 2010) |
|  |  |  |  |  |
| KPNA3 | 13 | 50273442 | 50367057 | Schizophrenia (Morris et al. 2012; Wei and Hemmings 2005; Zhang et al. 2006) |
|  |  |  |  |  |
| RNASEH2B | 13 | 51483813 | 51530901 | Aicardi-Goutières syndrome (Kind et al. 2014; La Piana et al. 2016; Rice et al. 2007) |
|  |  |  |  |  |
| ATP7B | 13 | 52506805 | 52585630 | Wilson Disease (de Bie et al. 2007; Dening Tr 1989) |
|  |  |  |  |  |
| SUGT1 | 13 | 53226830 | 53262433 | Alzheimer’s Disease (Spiechowicz et al. 2006) |
|  |  |  |  |  |
| PCDH8 | 13 | 53418108 | 53422775 | Synaptic Function (Hilschmann et al. 2002; Yamagata et al. 1999; Yasuda et al. 2007) |
